# Supplementary material for: Freshwater lake to salt-water sea causing widespread hydrate dissociation in the Black Sea
Source: Nat Commun. 2018 Jan 9;9:117. doi: 10.1038/s41467-017-02271-z (PMC5760725; doi:10.1038/s41467-017-02271-z)
Supplement: Supplementary file 1 — Supplementary Information [file 41467_2017_2271_MOESM1_ESM.pdf]

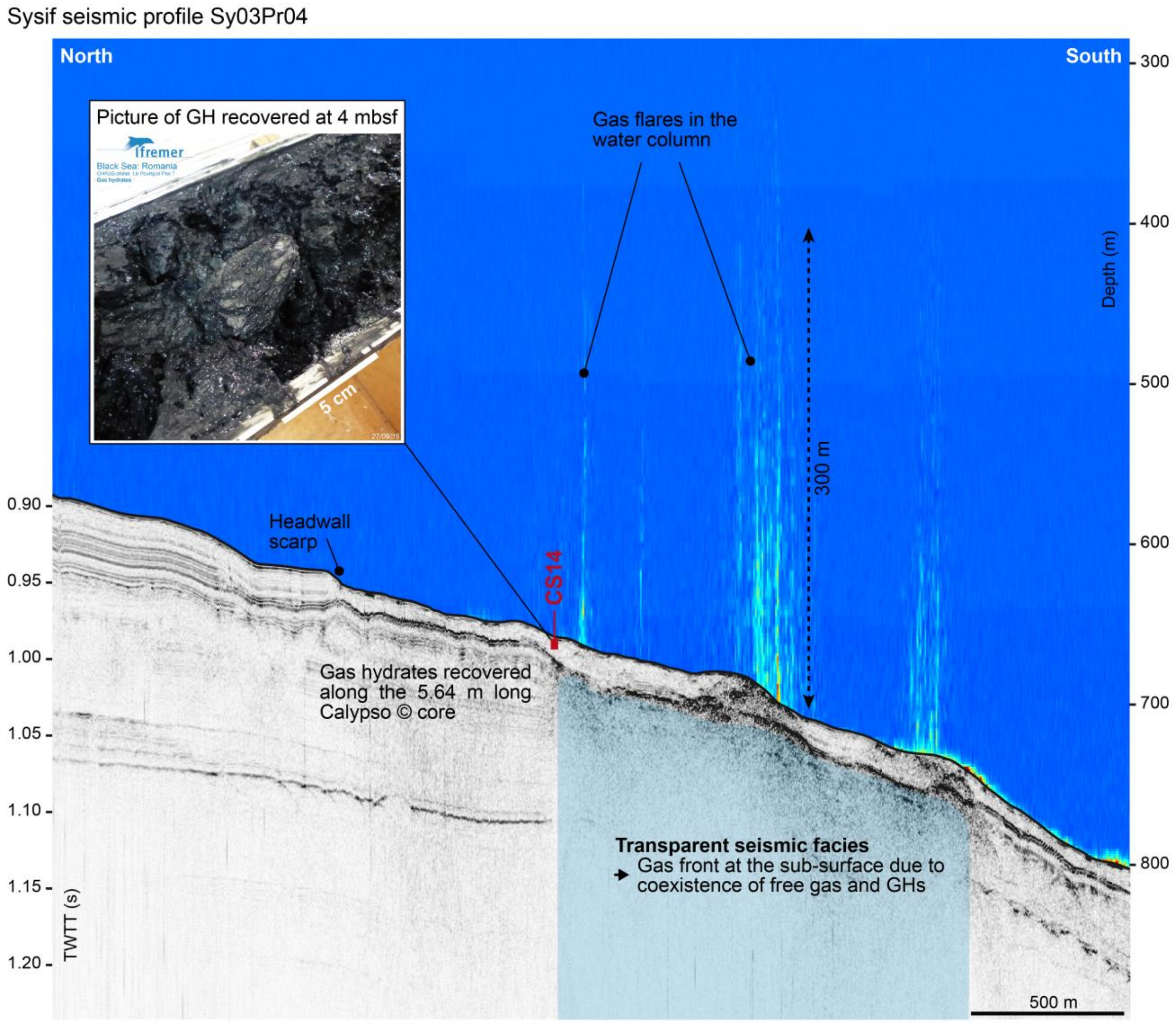

Supplementary Figure 1: Methane hydrate recovered at the seabed. The interpretation of a VHR seismic section (acquired with the deep-towed Sysif multi-channel system) shows a gas shadow which matches positively with occurrence of GH into the first 6 meters below seafloor (mbsf) of sediment and free gas in the water column. The processed water column echogram along the Sysif line shows the location of the gas flares inside the current GHSZ right above a fault system. The location of the CS14 core is indicated in the Supplementary Figure 2.

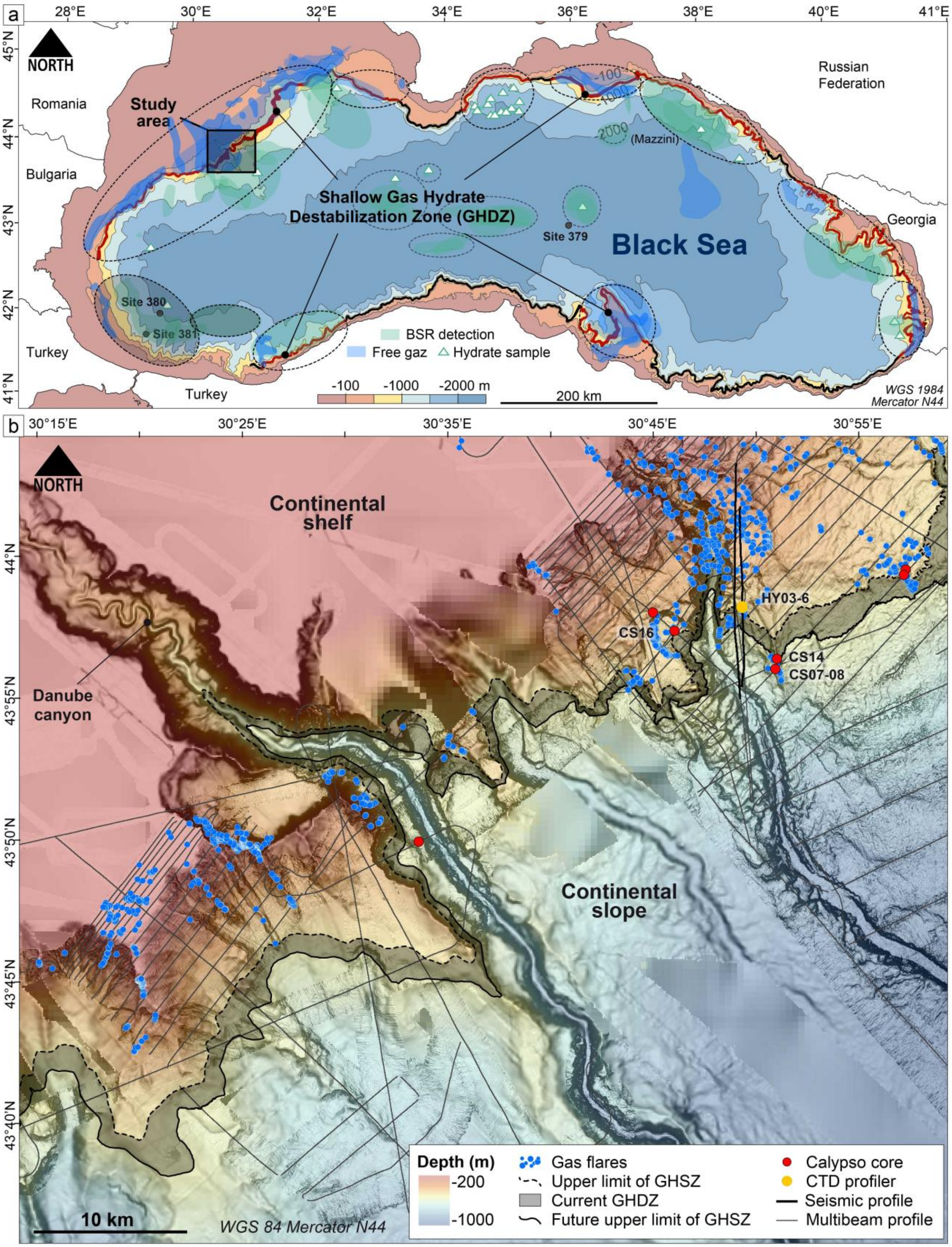
a
28°E
30°E
32°E
34°E
36°E
38°E
40°E
41°E
45°N
44°N
43°N
42°N
41°N
NORTH
Romania
Bulgaria
Turkey
Turkey
Georgia
Russian Federation
Study area
Shallow Gas Hydrate Destabilization Zone (GHDZ)
Black Sea
Site 379
Site 380
Site 381
(Mazzini)
-100
-1000
2000
BSR detection
Free gaz
Hydrate sample
-100
-1000
-2000 m
200 km
WGS 1984
Mercator N44
b
30°15'E
30°25'E
30°35'E
30°45'E
30°55'E
44°N
43°55'N
43°50'N
43°45'N
43°40'N
NORTH
Continental shelf
Continental slope
Danube canyon
HY03-6
CS16
CS14
CS07-08
Depth (m)
-200
-1000
Gas flares
Upper limit of GHSZ
Current GHDZ
Future upper limit of GHSZ
Calypso core
CTD profiler
Seismic profile
Multibeam profile
10 km
WGS 84 Mercator N44

Supplementary Figure 2: Gas hydrate province beyond 660 m water depth on the Romanian margin of the Black Sea. (**a**) The GHDZ (the black zone) close to the seafloor denotes the area where we suspect the reduction of the gas hydrate stability zone due to salinity diffusion through the sediment. By considering only areas where free gas/gas hydrates have been recovered or inferred[1-15] (areas in blue and green), we isolated the GHDZ (red zone) corresponding to the area where the GH zones are expected to decompose in the future (5000 yrs). As shown by Naudts *et al.* (2006) and Riboulot *et al.* (2017), the presence of free gas, detected in seismic data and/or in the water column close to the pinch out of the GHSZ, is an evidence of the presence of gas hydrates in deeper areas that play the role of buffer hampering the gas to reach the seafloor. We take into account this result to make the cartography of the occurrence of gas hydrates in the Black Sea; (**b**) The dark grey zone between the current and future landward terminations of the GHSZ denotes the area where the GH are expected to decompose in the future due to salinity diffusion through the sediment. The map shows the location of the seismic profiles, the cores and the CTD-rosette profile used in the present study. The gas flares (blue points) are detected during the GHASS cruise. They are observed within the shipborne RESON 7150 multibeam swath along the vessel track indicated by fine grey lines.

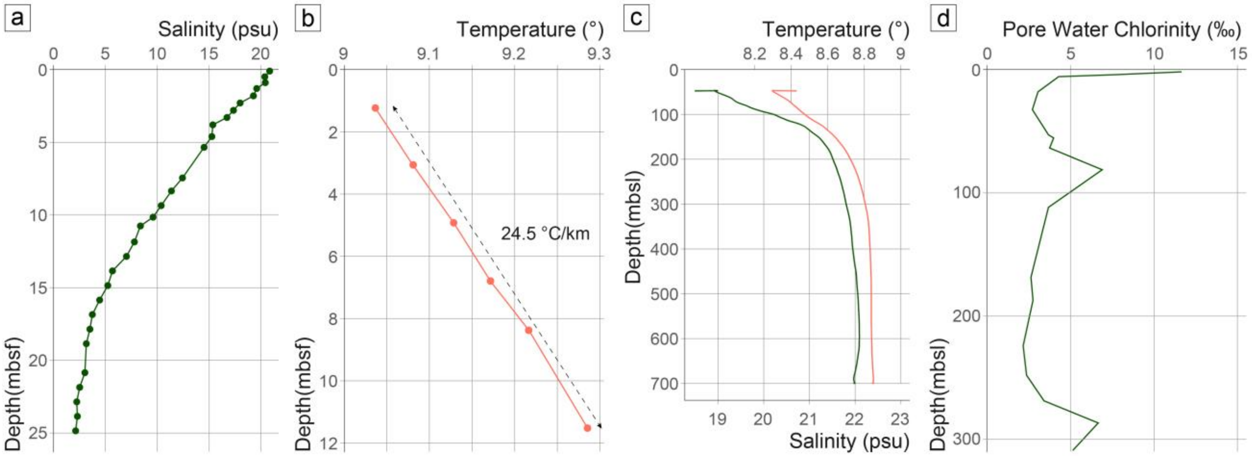

Supplementary Figure 3. *In situ* measurement profiles acquired during GHASS cruise and DSDP cruise. (**a**) Salinity profile within sedimentary column measured on a 25 m core CS16 (location of the core in Supplementary Fig. 1), (**b**) In situ geothermal gradient measured at the location of the CSF 04 is in agreement with the geothermal gradient calculated using the depth of the BSR in the Black Sea[8], (**c**) Water column salinity and temperature profiles at the location of the Hy03-Bat06-07 (location of the profile in Supplementary Fig. 1), (**d**) Chlorinity of the pore water at the DSDP hole 379A in the central plain of the Black Sea (modified from Merey and Sinayuc, 2016[13] and Calvert and Batchelor, 1978[16])

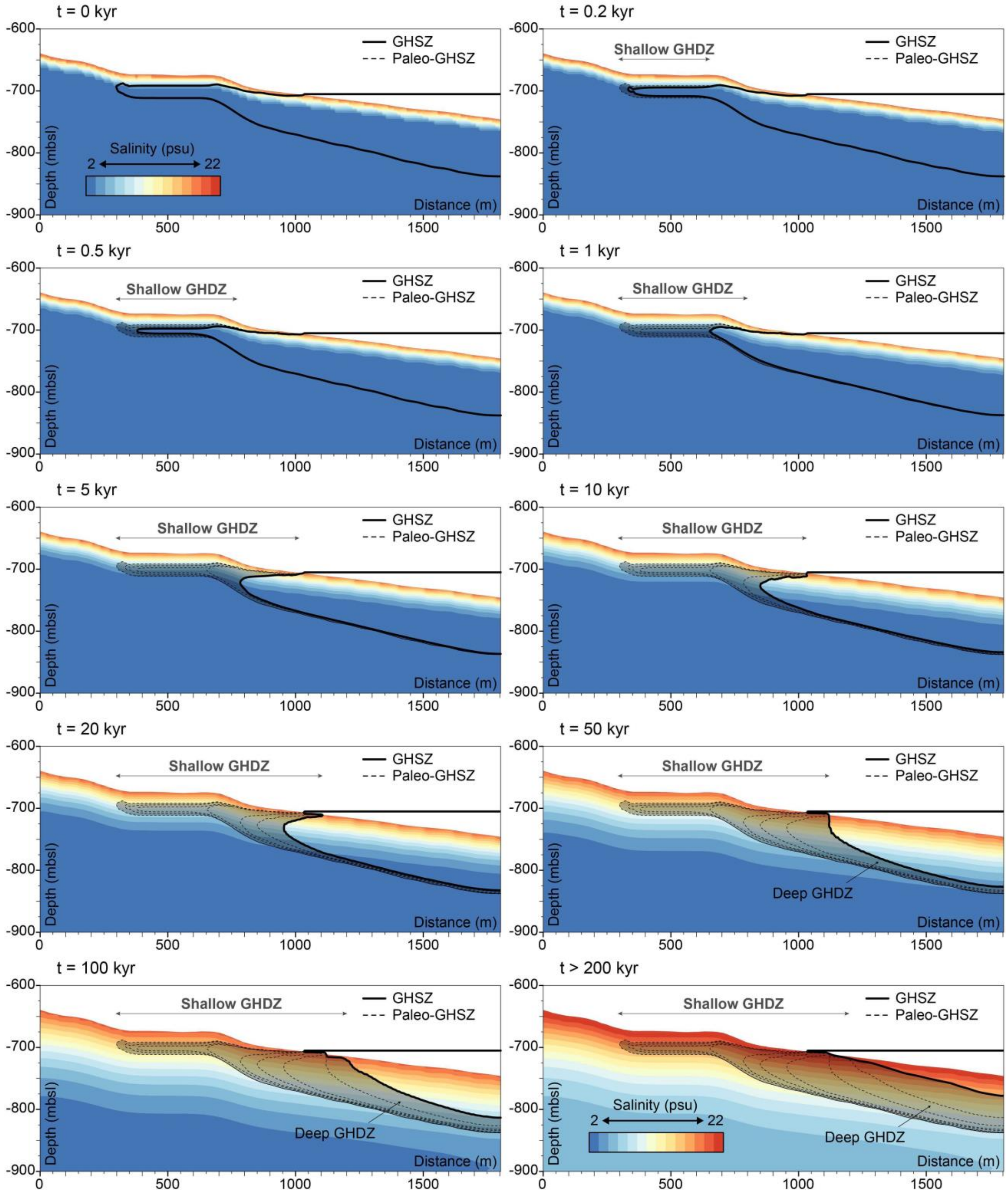

Supplementary Figure 4. Simulation of the evolution of the GHSZ over time. The results of the simulation using the two-dimensional numerical model developed by Sultan *et al.*[26] are presented through

10 steps of the calculation (t = 0, 0.2, 0.5, 1, 5, 10, 20, 50, 100 and >200 kyr). The landward termination of the GHSZ evolve over time. Hydrate deposit, the grey area, named “Gas Hydrate Destabilization Zone” (GHDZ) is currently undergoing dissociation caused by salt diffusion through sediments. This simulation is performed using the shallow part of the seismic profile presented in Fig. 2.

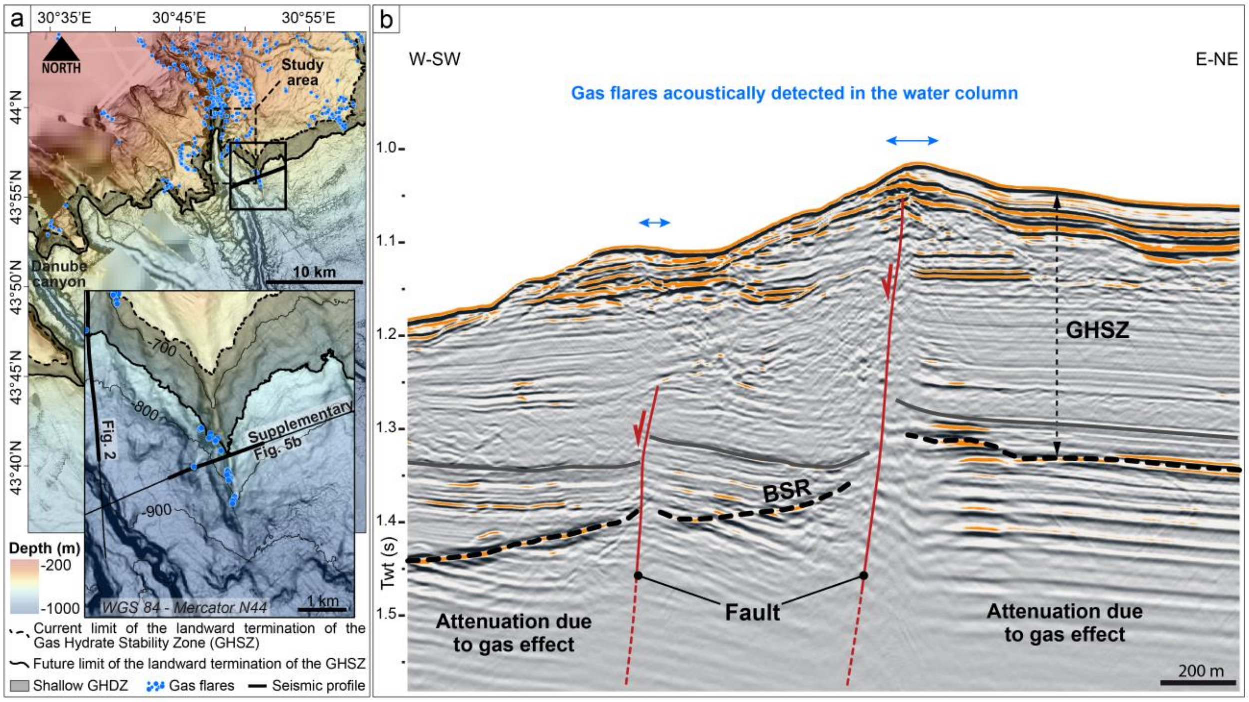

Supplementary Figure 5. Gas flares within the GHSZ. (**a**) Bathymetric map, acquired during the 2015 GHASS cruise, showing the study area with the location of seismic profiles presented in the supplementary figure 5b. The dark grey zone between the current and future landward terminations of the GHSZ denotes the area where the GH are expected to decompose in the future due to salinity diffusion through the sediment. The gas flares (blue points) are detected during the GHASS cruise. (**b**) HR seismic section show the presence of the BSR identified in the study area. The high attenuation of seismic wave under the BSR reveals the presence of free gas. Two normal faults are identified right under the gas flares acoustically detected in the water column.

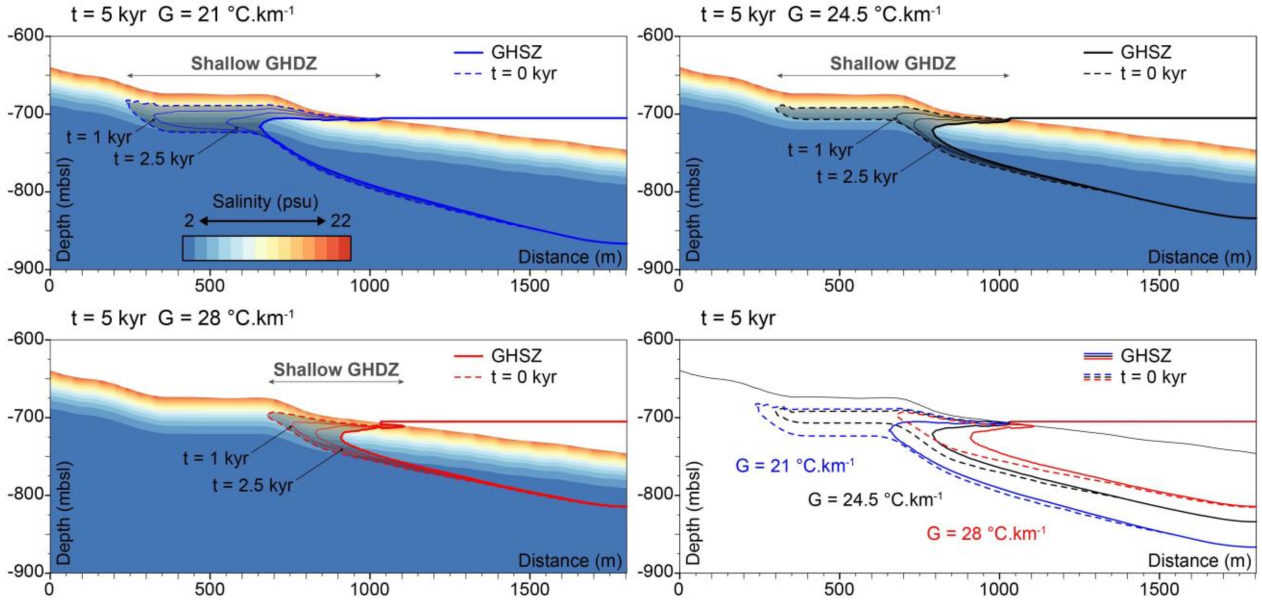

Supplementary Figure 6. Simulation of the evolution of the GHSZ over time by changing the geothermal gradient. We conducted two other numerical calculations, in addition to that presented in this study, to examine the impact of the geothermal gradient in GHSZ location and evolution over time. An increase of the geothermal gradient will induce a reduction of the GHSZ and the GHDZ while a decrease will increase the thickness of the GHSZ and the volume of hydrate deposit that currently dissociate.

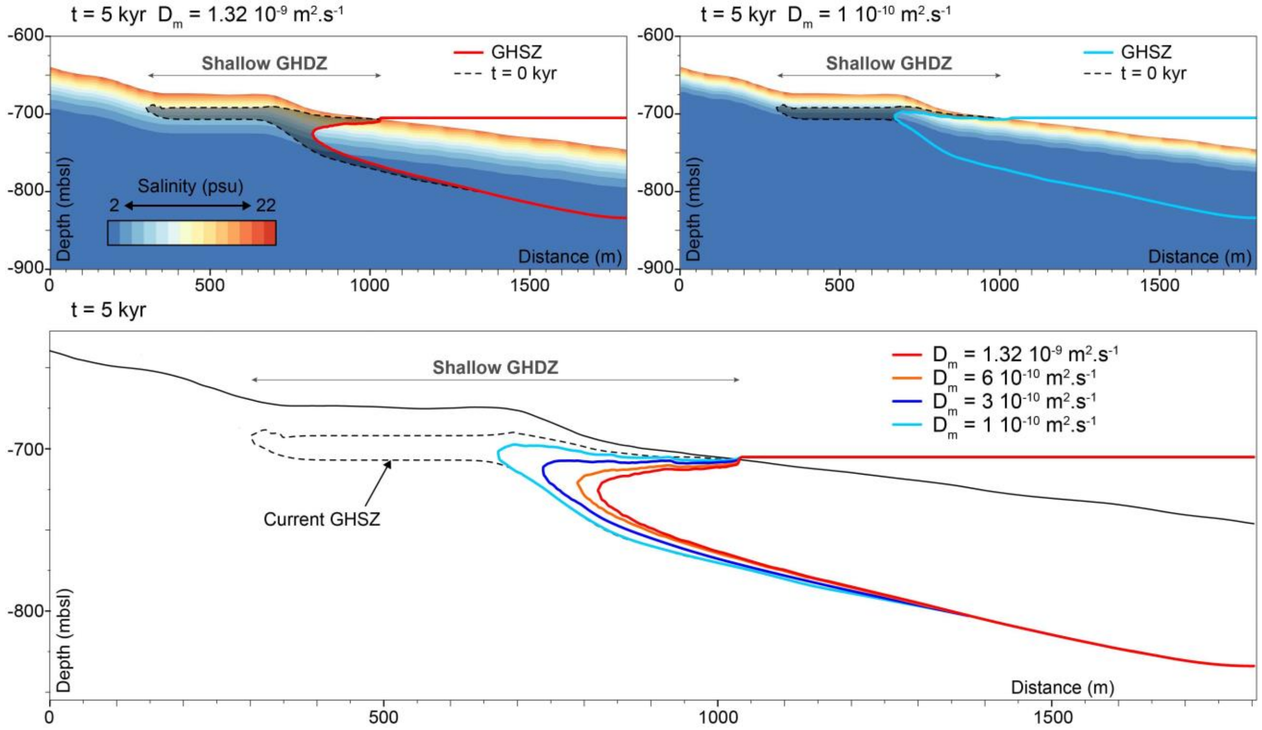

Supplementary Figure 7. Simulation of the evolution of the GHSZ over time by changing the chloride diffusion coefficient. We conducted three other numerical calculations, in addition to that presented in this study, to examine the impact of the chloride diffusion coefficient in GHSZ evolution over time. We have represented the results of the calculations in the same profile. Each colour line is the GHSZ in 5 kyr for different chloride diffusion coefficients. The impact of the chloride diffusion coefficient is significant but the gas hydrates within the shallow GHDZ will dissociate in all the cases in the next 5000 kyr.

Supplementary Note 1. Location of the gas flares. Most of gas flares recorded during the GHASS cruise are outside the GHSZ. Some gas flares are observed within the GHSZ. (1) Those inside the GHDZ could be explained by the salinization of sediment generating GH dissociation and thus free gas expulsion in the water column. (2) Those outside the GHDZ are recorded at around 800 mbsl right above a fault system (Supplementary Fig. 5). The fault system with a normal fault reaching the seafloor within the GHSZ provide a vertical gas migration pathway (Supplementary Fig. 5). It provide a permeable discontinuity where the free gas can migrate throughout the GHSZ. The fault location, in agreement with the gas flares could explain the presence of these gas seeps inside the GHSZ and outside the GHDZ. The indirect evidence of the presence of free gas under the BSR, the large amounts of gas flares outside the GHSZ and the lack of gas flares inside the GHSZ suggest GH play the role of impermeable caprock and trap free gas under the base of their stability zone.

Supplementary Note 2. Parametric study of the GHSZ evolution. We conducted 4 other numerical calculations (Supplementary Figs. 6 and 7), in addition to that presented in the article, to examine the impact of the geothermal gradient and the salt diffusion coefficient on GHSZ evolution over time. Despite a significant impact of these two parameters in simulation of evolution of the GHSZ, the gas hydrates within the shallow GHDZ currently dissociate and will dissociate in all the cases in the next 5000 kyr.

Supplementary References

1 Ergün, M., Dondurur, D. & Çifçi, G. Acoustic evidence for shallow gas accumulations in the sediments of the Eastern Black Sea. *Terra Nova* **14**, 313-320 (2002).

2 Vassilev, A. & Dimitrov, L. Model evaluation of the Black Sea gas hydrates. *Comptes Rendus de l'Academie bulgare des Sciences* **56**, 3-15 (2003).

3 Vassilev, A. Optimistic and pessimistic model assessments of the black sea gas hydrates. *Comptes Rendus de l'Academie bulgare des Sciences* **59**, 543-550 (2006).

4 Poort, J., Vassilev, A. & Dimitrov, L. Did postglacial catastrophic flooding trigger massive changes in the Black Sea gas hydrate reservoir? *Terra Nova* **17**, 135-140 (2005).

5 Riboulot, V., Cattaneo, A., Scalabrin, C. & Gaillot, A. Control of the geomorphology and gas hydrate extent on widespread gas emissions offshore Romania. *BSGF-Earth Sciences Bulletin*, ***188***(4), 26 (2017).

6 Popescu, I. *et al.* Multiple bottom-simulating reflections in the Black Sea: potential proxies of past climate conditions. *Marine Geology* **227**, 163-176 (2006).

7 Popescu, I., Lericolais, G., Panin, N., De Batist, M. & Gillet, H. Seismic expression of gas and gas hydrates across the western Black Sea. *Geo-Marine Letters* **27**, 173-183 (2007).

8 Zander, T. *et al.* On the origin of multiple BSRs in the Danube deep-sea fan, Black Sea. *Earth and Planetary Science Letters* **462**, 15-25 (2017).
9 Dondurur, D., Küçük, H. M. & Çifçi, G. Quaternary mass wasting on the western Black Sea margin, offshore of Amasra. *Global and Planetary Change* **103**, 248-260 (2013).
10 Pape, T., Bahr, A., Klapp, S. A., Abegg, F. & Bohrmann, G. High-intensity gas seepage causes rafting of shallow gas hydrates in the southeastern Black Sea. *Earth and Planetary Science Letters* **307**, 35-46 (2011).
11 Mazzini, A. *et al.* Complex plumbing systems in the near subsurface: geometries of authigenic carbonates from Dolgovskoy Mound (Black Sea) constrained by analogue experiments. *Marine and Petroleum Geology* **25**, 457-472 (2008).
12 Naudts, L. *et al.* Geological and morphological setting of 2778 methane seeps in the Dnepr paleo-delta, northwestern Black Sea. *Marine Geology* **227**, 177-199 (2006).
13 Merey, S. & Sinayuc, C. Analysis of the Black Sea sediments by evaluating DSDP Leg 42B drilling data for gas hydrate potential. *Marine and Petroleum Geology* **78**, 151-167 (2016).
14 Merey, S. & Sinayuc, C. Investigation of gas hydrate potential of the Black Sea and modelling of gas production from a hypothetical Class 1 methane hydrate reservoir in the Black Sea conditions. *Journal of Natural Gas Science and Engineering* **29**, 66-79 (2016).
15 Klaucke, I. *et al.* Acoustic investigation of cold seeps offshore Georgia, eastern Black Sea. *Marine Geology* **231**, 51-67 (2006).
16 Calvert, S. E. & Batchelor, C. H. Major and minor element geochemistry of sediments from Hole 379A, Leg 42B, Deep-Sea Drilling Project. *Initial reports of the deep sea drilling project* **42**, 527-541 (1978).
